# Optimization of sonication for preserving the biochemical and physicochemical attributes of pomegranate arils during storage

**DOI:** 10.1016/j.ultsonch.2026.107763

**Published:** 2026-02-07

**Authors:** Isa Hazbawi, Hamed Etezadi

**Affiliations:** a Department of Biosystems Engineering, Faculty of Agriculture, Lorestan University, Khorramabad, Iran; b Department of Bioresource Engineering, Faculty of Agricultural and Environmental Sciences, McGill University, Montreal, Canada

**Keywords:** Acoustic cavitation, Non-thermal processing, Postharvest preservation, Shelf-life extension, Process optimization

## Abstract

Pomegranate arils rapidly experience quality deterioration and a decline in bioactive compounds during storage. This study aimed to develop and optimize an effective non-thermal technology by evaluating the efficacy of ultrasound treatment in preserving the biochemical and physicochemical attributes of pomegranate arils using response surface methodology (RSM). The effects of ultrasound time (4–14 min) and storage duration (0–16 days) on key quality indicators, including total phenol content (TPC), antioxidant activity (AOA), total anthocyanin content (TAC), titratable acidity (TA), and weight loss (WL), were assessed. Results indicated that controlled increases in ultrasound exposure helped maintain bioactive compounds, whereas quality loss was primarily dependent on storage duration. The optimal conditions were determined as 12.3 min of ultrasound and 7.1 days of storage, under which AOA of 71.58%, TA of 2.3%, TAC of 155.16 mg 100 g^−1^, and TPC of 133.82 mg 100 g^−1^ were predicted, along with minimal WL of 1.32%. Experimental validation confirmed the accuracy of the model and demonstrated that ultrasound can effectively mitigate quality decline in pomegranate arils. The findings suggest that this non-thermal technology provides a sustainable and practical approach for postharvest quality management and the development of advanced storage systems, particularly within cold chains for sensitive products.

## 1 Introduction

Modern food-processing technologies, particularly non-thermal methods, have been introduced in recent years as effective strategies to extend shelf life while preserving the nutritional and bioactive components of fresh products. Although conventional thermal treatments effectively reduce microbial loads, they may degrade sensitive bioactive compounds and reduce nutritional quality, thereby highlighting the need for milder preservation approaches [1]. In contrast, non-thermal technologies such as ultrasound, high hydrostatic pressure, and supercritical carbon dioxide, through minimal heat input and short processing times, allow the preservation of heat-sensitive attributes in fresh commodities [2], [3]. These technologies also enhance processing efficiency while reducing nutritional damage and improving both performance and quality in agricultural products.

Among these technologies, the application of ultrasound (US) has attracted special attention due to its minimally invasive nature, high efficiency, and favorable operational cost. Ultrasound induces phenomena such as cavitation, localized microstreaming, and shear stresses, which lead to controlled modifications in tissue structure, enzymatic activity, and microbial behavior of fresh products [4], [5]. Research evidence has shown that ultrasound treatments can prevent the loss of valuable constituents such as anthocyanins, ascorbic acid, and phenolics during storage, thereby improving the nutritional stability of fruits [6], [7]. This capability has made ultrasound a promising option for postharvest management of highly perishable commodities.

Pomegranate (*Punica granatum* L.) is one of the most significant fruits rich in bioactive compounds and antioxidants, and it possesses high nutritional and medicinal importance due to its anti-inflammatory and anticancer properties [8], [9]. However, pomegranate arils, because of their high moisture content, susceptibility to mechanical injury, and rapid microbial spoilage, undergo quality deterioration within a short time [10], [11]. This sensitivity limits the utilization of fresh pomegranate arils and underscores the growing need for effective, minimally invasive preservation strategies.

Despite numerous studies on the effects of ultrasound on fresh fruits, limited information is available regarding the influence of this technology on the biochemical and physicochemical characteristics of pomegranate arils during storage [12]. In particular, few investigations have simultaneously evaluated time, intensity, and temperature within a systematic experimental-design framework, even though these variables play a critical role in retaining acidity, phenolic content, and antioxidant activity [13], [14]. Consequently, a substantial knowledge gap remains regarding the optimal ultrasound conditions required to preserve the quality of pomegranate arils.

Advanced statistical techniques such as response surface methodology (RSM) can serve as powerful tools for the design, analysis, and optimization of postharvest treatments. The application of RSM enables the assessment of interactive effects among ultrasound variables and supports the prediction of biochemical and physiological responses, thereby facilitating the determination of optimal and cost-effective conditions for extending shelf life and preserving the quality of fresh products [15], [16]. Previous studies have shown that RSM is capable of adjusting ultrasound intensity and duration in various commodities while improving their nutritional and sensory attributes [17], [18].

With increasing demand for fresh and ready-to-eat products, the need for mild technologies to maintain the quality of pomegranate arils has become more evident. The present study was designed to optimize ultrasound conditions for preserving bioactive compounds, reducing weight loss, and extending the storage duration of pomegranate arils. Using RSM, the simultaneous effects of ultrasound time and storage duration were analyzed, and optimal conditions were identified to support the development of practical strategies for postharvest management.

## 2 Materials and methods

### 2.1 Fruit harvesting, experimental conditions, and storage

Pomegranate fruits of the Saveh cultivar at full maturity were collected from orchards in Khorramabad (Iran) and were immediately transferred to the laboratory after detachment from the branches, where ultrasound treatments were applied. Samples were carefully selected, and only sound and uniform fruits were included in the process. They were washed with distilled water, dried, and manually arils were extracted. The arils were exposed to ultrasound waves at a frequency of 40 kHz (Backer device, model vCLEAN1-L6, Iran). The treatments were conducted for 4, 9, and 14 min at a constant temperature of 25°C.

**Fig. 1:**
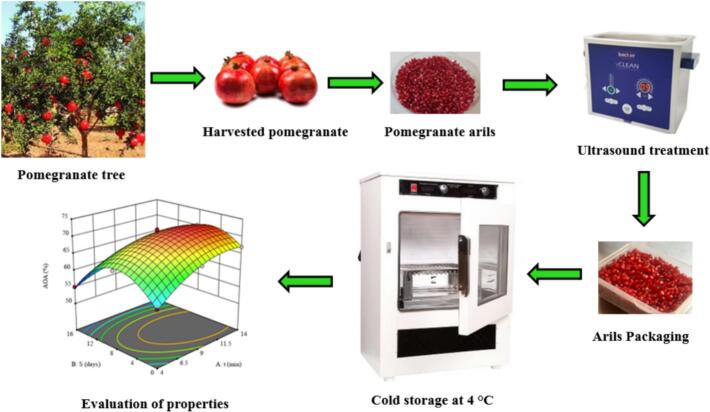
Schematic representation of the effects of ultrasound treatment on the biochemical and biophysical properties of pomegranate arils during storage.

After treatment, the arils were packaged in 50 g replicates in transparent polyethylene containers and were stored under controlled conditions (4°C and 85% relative humidity) to enable precise monitoring of their qualitative and nutritional changes. Control samples (non-ultrasound) were prepared and stored under identical conditions as the ultrasound-treated samples to enable reliable comparison of treatment effects. Sampling was carried out on days 0, 8, and 16 to allow accurate evaluation of the studied attributes (Fig. 1). The Saveh cultivar was selected due to its economic importance, desirable productivity, and significant contribution to export markets, and prominent status among native Iranian cultivars. The treatment duration and storage duration were defined based on previous findings and recommendations [17], [19] to ensure optimal preservation of the quality and nutritional value of pomegranate arils.

### 2.2 Determination of titratable acidity (TA)

For the determination of TA, a defined volume of pomegranate juice (10 mL) was mixed with 40 mL of distilled water to obtain a homogeneous, measurable sample. Then, 10 mL of the diluted solution was titrated against 0.1 N sodium hydroxide until the final pH reached 8.2. Phenolphthalein was used as an indicator to precisely identify the endpoint of the neutralization reaction by its color change. The TA of the samples was calculated based on the percentage of titratable acids and reported quantitatively [20].

### 2.3 Determination of total anthocyanin content (TAC)

The TAC was determined using the standard pH-differential method. For this purpose, 5 g of the homogenized fruit sample was centrifuged at 10,000 rpm for 20 min, and the resulting supernatant was filtered and diluted in two acidic buffers with pH values of 1.0 and 4.5. The absorbance of the solutions was then measured at 520 and 700 nm using a UV–Vis spectrophotometer (uv1100, Mapada Co, Shanghai, China) to quantify absorbance differences associated with structural changes in anthocyanin at the two pH levels. The TAC was calculated using Equation (1) and expressed as cyanidin-3-O-glucoside equivalents per 100 g of fresh weight [21].

(1) $$\mathrm{TAC}=[{\left(A520-A700\right)}_{pH1}-{\left(A520-A700\right)}_{pH4.5}]$$

Where the absorbance values at wavelengths of 520 nm (A520) and 700 nm (A700) are defined.

### 2.4 Determination of total phenol content (TPC)

The TPC of the samples was determined based on the gallic acid standard curve using the Folin–Ciocalteu chromogenic reaction. Absorbance was measured with a spectrophotometer at 725 nm to enhance analytical sensitivity and accuracy. The results were expressed as gallic acid equivalents in milligrams per 100 g of fresh weight [21].

### 2.5 Measurement of weight loss (WL)

To determine WL with precision, pomegranate arils were weighed on day zero and again at the end of each specified interval. The difference between the initial and final weights was calculated as the WL. The results were presented as percentages to allow comparative assessment and interpretation of weight changes during the experimental period [20].

### 2.6 Determination of antioxidant activity (AOA)

The AOA of the arils was evaluated using the DPPH free radical scavenging assay. One gram of fruit tissue was homogenized in 8 mL of acidified methanol (1% HCl) and extracted for 24 h in darkness. The samples were then centrifuged at 12,000 rpm for 20 min at 4°C. For AOA measurement, 50 μL of the supernatant was added to 1 mL of DPPH solution at a concentration of 6 × 10^-5^ mol·L^−1^, and the change in absorbance at 515 nm was recorded. The AOA was determined by calculating the percentage of DPPH radical inhibition using Equation (2) [21].

(2) $$AOA\;\left(\%\right)=\frac{Acontrol\;\_\;Asample}{Acontrol}\times 100$$

Where Acontrol represents the absorbance of the blank and Asample represents the absorbance of the sample.

### 2.7 Experimental design and data analysis

This study was conducted to optimize ultrasound treatment for maintaining quality and reducing WL of pomegranate arils during storage. Ultrasound exposure time (4, 9, and 14 min) and storage duration (0, 8, and 16 days) were examined as independent variables, and their effects on TA, WL, AOA, TAC, and TPC were evaluated. The data were analyzed using second-order regression within a RSM framework to establish quantitative relationships between treatment conditions and quality responses. Optimal ultrasound conditions were determined according to Equation (3) [22].

(3) $$Y=B_{0}+B_{1}X_{1}+B_{2}X_{2}+{B_{3}X_{1}X_{2}+B}_{4}X_{1}^{2}+B_{5}X_{2}^{2}$$

The response variable (*Y*) was modeled as a function of ultrasound time (*X_1_*) and storage duration (*X_2_*). Estimated coefficients—including the intercept (*B_0_*), main effects (*B_1_*, *B_2_*), interaction effect (*B_3_*), and nonlinear terms (*B_4_*, *B_5_*)—were used to evaluate the magnitude and direction of factor influences.

Simulation and optimization of ultrasound treatment for pomegranate arils were performed using response surface methodology and a central composite design (CCD) in Design-Expert 11. Independent variables were evaluated at a significance level of 0.05, and only significant factors were retained in the final regression model. Based on the data in Table 1 and considering operational constraints and research objectives, optimal treatment conditions were determined to simultaneously maximize biochemical attributes and minimize WL. Principal component analysis and Pearson correlation coefficients were computed using R software (version 2022) to assess statistical relationships and interdependencies among variables with high analytical clarity.

**Table 1:** Operational limits of variables and constraint requirements.

Parameter	Unit	Symbol	Min	Max	Target
Ultrasound time	min	t	4	14	In range
Storage duration	days	S	0	16	In range
Antioxidant activity	%	AOA	55.08	72.06	Maximum
Titratable acidity	%	TA	1.72	2.51	Maximum
Total phenol content	mg 100 g^−1^	TPC	112.28	135.04	Maximum
Total anthocyanin content	mg 100 g^−1^	TAC	140.14	157.15	Maximum
Weight loss	%	WL	0	3.32	Minimum

## 3 Results and discussion

### 3.1 Correlation analysis and principal component analysis of pomegranate aril traits

**Fig. 2:**
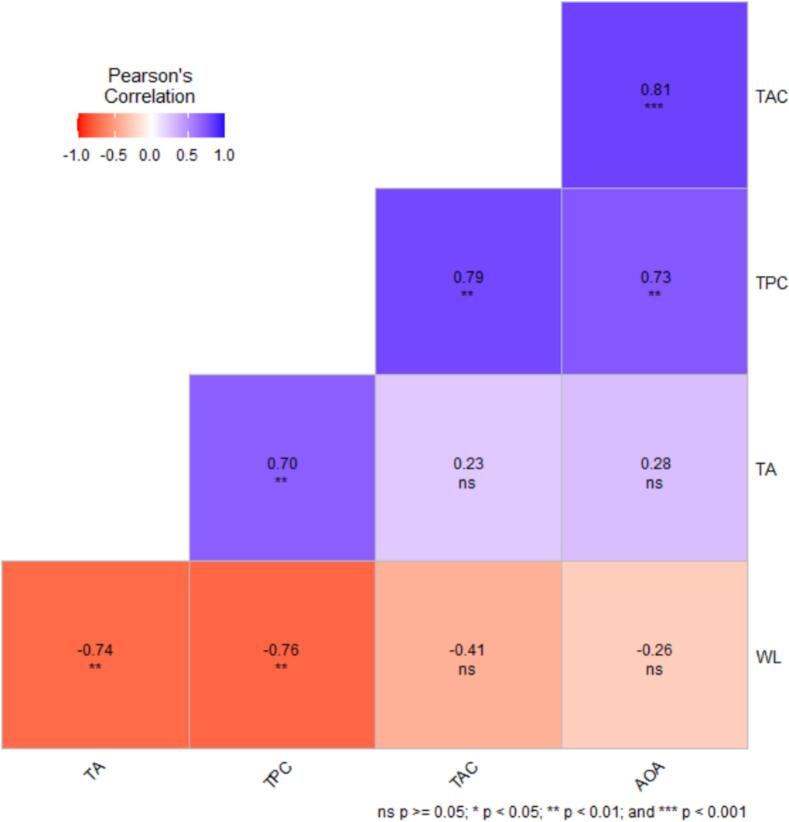
Pearson correlations among antioxidant activity (AOA), titratable acidity (TA), total anthocyanin content (TAC), weight loss (WL), and total phenol content (TPC) in ultrasound-treated pomegranate arils during storage.

The Pearson correlation matrix indicated that TA and TPC were negatively and significantly correlated with WL (p < 0.05, Fig. 2). This finding suggests that increases in phenolic compounds and acidity were associated with reduced WL during ultrasound treatment and storage. Moreover, TAC was positively and significantly correlated with TPC, and particularly with AOA (p < 0.05). This association confirms that TAC plays a key role in the overall antioxidant capacity of pomegranate arils.

TPC was also positively and significantly correlated with AOA and TA (p < 0.05), indicating a close relationship between phenolic compounds and antioxidant performance. By contrast, TAC and AOA showed weak and non-significant correlations with WL and TA (p > 0.05), reflecting the relative independence of these traits under ultrasound treatment and storage conditions. These results highlight that each bioactive attribute may respond differently to processing conditions, providing valuable insight for optimizing ultrasound time and intensity to maintain fruit quality. Overall, the bioactive components and physical properties of pomegranate arils exhibited distinct yet interactive roles throughout ultrasound processing and storage. These findings may offer useful guidance for designing optimal processing conditions and developing industrial storage systems.

**Fig. 3:**
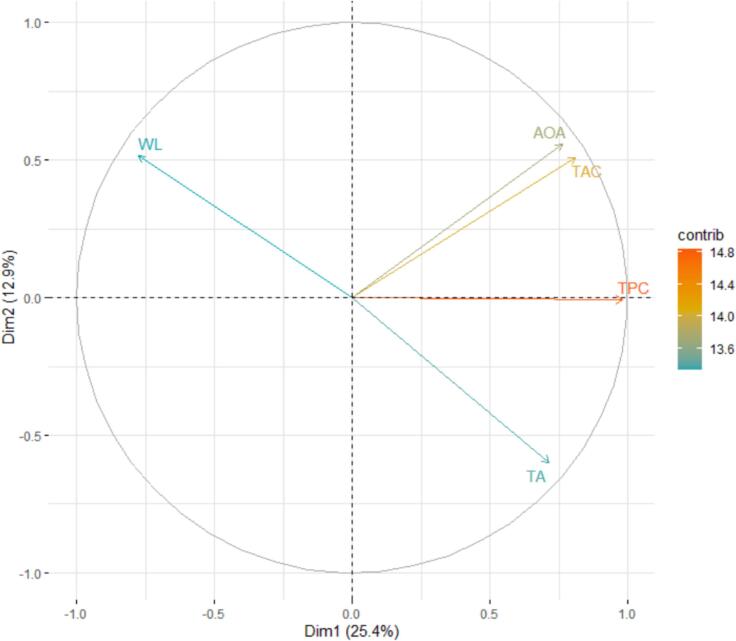
Principal component analysis (PCA) of the biochemical and physiological characteristics of pomegranate arils under ultrasound treatment and storage.

The biplot generated from the principal component analysis revealed that PC1 and PC2 accounted for 25.4% and 12.9% of the total variance, respectively, reflecting the variations in pomegranate aril traits influenced by ultrasound time and storage duration (Fig. 3). The vectors of antioxidant and phenolic traits (AOA, TAC, and TPC) were positioned mainly along the positive direction of PC1, and their coordinated shifts suggested similar responses to the treatments. In contrast, WL and TA were located in negative directions, exhibiting patterns distinct from the other traits. The wide angle between the WL vector and the antioxidant traits indicated a negative correlation, and the placement of TA in the opposite quadrant showed that higher acidity was associated with reduced antioxidant attributes. Sample clustering within the two-component space further demonstrated that samples with high AOA, TAC, and TPC were concentrated in the positive region of PC1, whereas samples with high WL or TA were grouped in opposite directions. This pattern clearly illustrates the effects of ultrasound time and storage duration on the quality of pomegranate arils.

### 3.2 Determinants of pomegranate arils quality under ultrasound treatment during storage

Regression models developed with high R^2^ values (0.948–0.991) indicated an adequate fit to the data and a strong ability to explain variations in the biochemical and physiological attributes of pomegranate arils (Table 2). ANOVA results showed that both ultrasound exposure time and storage duration, together with their interaction effects, exerted significant influences on the responses (p < 0.05), confirming the importance of process–storage interplay. The nonlinear patterns observed in the quadratic coefficients suggested that the response of bioactive compounds to ultrasound exhibited a complex nature, in which ultrasound intensity enhanced compound retention only up to a certain threshold. It was found that a moderate increase in ultrasound time could reduce the degradation of phenolic, antioxidant, and anthocyanin compounds, while quality losses were mainly governed by the length of the storage duration. These findings indicate that concurrent optimization of ultrasound parameters and storage duration can serve as an effective strategy for preserving the nutritional quality of pomegranate arils in postharvest systems.

**Table 2:** Nonlinear regression fitting to experimental responses.

Parameter	Model	R^2^
AOA	70.82 + 2.65 t – 3.55S – 2.00tS – 3.33t^2^ – 8.63S^2^	0.99
TA	2.13 + 0.11 t – 0.26S + 0.11tS + 0.19t^2^ – 0.08S^2^	0.991
TPC	131.91 + 4.31 t + 6.13S + 3.45tS – 3.06t^2^ – 3.58S^2^	0.961
TAC	156.34 + 1.85 t – 2.32S + 2.52tS – 5.64t^2^ – 4.12S^2^	0.984
WL	1.53 – 0.45 t + 1.22 T – 0.43tS + 0.49t^2^ – 0.63S^2^	0.948

t: ultrasound time (min); S: storage duration (days); R^2^: coefficient of determination

### 3.3 AOA

**Fig. 4:**
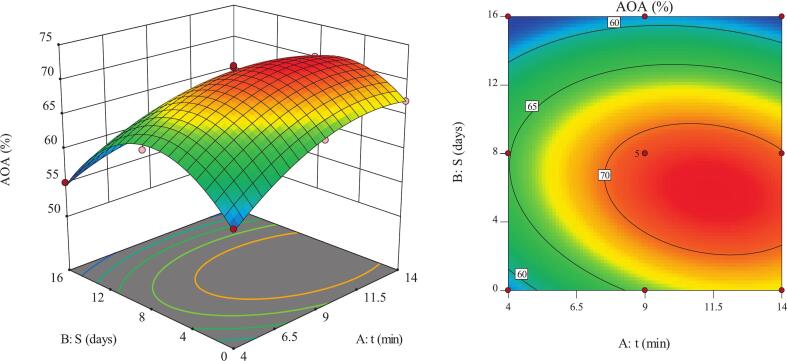
Interactive effects of ultrasound time (t) and storage duratin (S) on AOA of pomegranate arils.

The AOA is one of the principal criteria for assessing the antioxidant capacity and biochemical stability of fruits. Variations in this index are used to indicate the nutritional status and the fruit’s resistance to oxidative stress, providing valuable insights into its biological quality and the performance of its active constituents [21]. The response surface analysis of AOA in pomegranate arils revealed the interactive effect of ultrasound treatment time and storage duration (Fig. 4). A downward concave surface indicated the presence of an optimum point within the experimental domain. The highest AOA value (72.06%) was obtained at medium to long ultrasound treatment times and approximately 8 days of storage. This enhancement was likely caused by a more efficient release of antioxidant compounds trapped within the cellular matrix and improved stability of these compounds under optimal conditions. Conversely, the lowest AOA value (55.08%) occurred under short ultrasound exposure (4 min) combined with prolonged storage (16 days), which may be attributed to insufficient extraction of active compounds and their oxidative degradation during storage. The control samples exhibited an AOA of 48.7% on day 16, representing approximately 6.4 percentage points lower antioxidant activity than the ultrasound-treated samples (∼13% relative increase), indicating improved retention of antioxidant compounds during prolonged storage.

The results showed that during short storage durations, increasing ultrasound time led to a rapid rise in AOA (Fig. 4). This finding supports the role of mechanical energy and cavitation phenomena in releasing phenolic compounds and anthocyanins [23], [24]. However, at longer storage durations, the slope of AOA enhancement decreased. This decline was likely associated with the degradation or polymerization of a fraction of the bioactive compounds prior to sonication [25], [26]. The results aligned with the findings of Pan et al., [27] and Lu et al., [6], who reported that short-term ultrasound exposure increases phenolics and AOA, whereas prolonged treatments may exert detrimental effects. Recent studies further confirm this trend, showing that controlled ultrasound treatments significantly enhance antioxidant capacity and phenolic stability in plant products, with increases of 22–30% in radical-scavenging activity and anthocyanin retention during storage [28], [29]. Therefore, changes in AOA were influenced by the balance between compound release through cavitation and the reduction in their concentration due to degradation or polymerization reactions. This observation highlights the importance of identifying an optimal ultrasound time to maximize antioxidant capacity and preserve bioactive constituents [12], [30].

Storage duration also had a significant effect on AOA variations in pomegranate arils (Fig. 4). The initial increase in AOA during the early days was likely related to the activation of hydrolytic enzymes and the release of phenolic compounds bound to sugars or proteins [31], [32]. However, with extended storage—particularly under more intense ultrasound treatment—cellular structure disruption induced by cavitation accelerated the exposure of bioactive constituents to oxidation. This phenomenon intensified the decline in AOA [33], [34]. Similar studies have demonstrated that ultrasound can increase phenolic and vitamin content in the short term, while prolonged storage compromises their stability [6], [35]. Accordingly, the interaction between storage duration and ultrasound intensity, through mechanisms such as phenolic compound release and cavitation effects, plays a decisive role in determining AOA variability and bioactive stability [36], [37].

### 3.4 TA

**Fig. 5:**
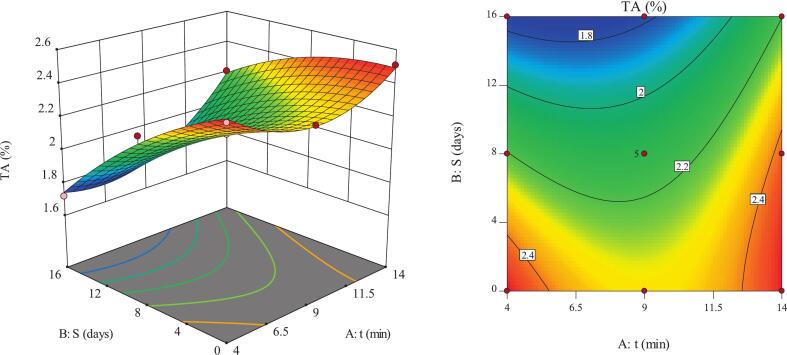
Response surface of TA in pomegranate arils as affected by ultrasound time during storage.

TA is recognized as a key postharvest quality index in fresh fruits and plays an important role in flavor, consumer appeal, and overall shelf life [20]. The three-dimensional RSM analysis indicated that the TA of pomegranate arils increased as ultrasound time increased, whereas prolonged storage led to a gradual decline (Fig. 5). The highest TA value (2.51%) was recorded under long-time ultrasound before storage, likely due to enhanced solubility and accessibility of organic acids induced by cavitation and partial cell wall disruption. In contrast, the lowest TA value (1.72%) occurred under short-time ultrasound combined with long storage, which may reflect the metabolic consumption of organic acids during respiration throughout the storage duration. The control samples exhibited a TA value of 1.17% on day 16, representing approximately 0.55 percentage points lower acidity than the ultrasound-treated samples (∼47% relative difference), indicating better retention of organic acids under ultrasound processing. These patterns were statistically significant (p < 0.05), suggesting that TA is simultaneously influenced by the physicochemical mechanisms of ultrasound and storage-related biochemical changes. Based on these findings, precise adjustment of ultrasound time can serve as an effective approach for managing flavor-related quality in postharvest pomegranate arils.

The results showed that TA declined significantly as storage duration increased (Fig. 5). This trend was mainly attributed to the respiratory consumption of organic acids and their involvement in biosynthetic pathways. The reduction in TA was more pronounced in short-time ultrasound treatments, as limited extraction of organic acids and better tissue integrity resulted in their faster utilization in metabolic processes [38]. In contrast, longer ultrasound exposure led to better retention of TA, possibly due to the gradual release of more stable acidic compounds or mild structural alterations, consistent with findings by Shi et al., [13] and Zhou et al., [39]. Recent studies further support these findings, showing that ultrasound treatment can enhance titratable acidity and maintain bioactive compounds during storage, with longer sonication times generally resulting in better preservation of organic acids in pomegranate arils [30], [40]. These results align with previous research and highlight that targeted ultrasound application can effectively preserve acidity and improve the quality of stored pomegranate arils [10], [41].

Increasing ultrasound time elevated the TA of pomegranate arils during both short- and long-term storage (Fig. 5). The increase was more pronounced during short-term storage, indicating the release of entrapped organic acids caused by cell wall disruption [13]. During longer storage, natural reductions in free acids moderated the ultrasound-induced increase, although the extraction effect remained dominant [42]. The mechanisms involved included cell structure disintegration, enhanced membrane permeability, and metabolic defense activation, which facilitated acid release and supported the stability of bioactive compounds [43]. This behavior aligns with the study’s objectives and the recognized role of ultrasound in improving pomegranate aril quality during storage. The findings agree with previous reports in other fruits, demonstrating that ultrasound can maintain quality and bioactive constituents throughout storage [6], [7]. Overall, the results highlight the potential of ultrasound to enhance the short-term storage quality of pomegranates.

### 3.5 TPC

**Fig. 6:**
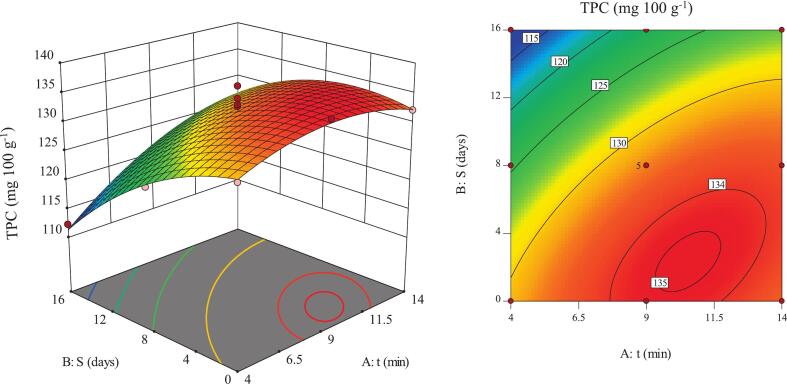
Interactive effects of ultrasound time and storage duratin on TPC of pomegranate arils.

TPC is widely used as an indicator of antioxidant capacity and bioactive properties in fruits. Phenolic compounds play a central role in enhancing bioactivity and improving the nutritional and health-related quality of fruits [20]. The response surface analysis showed that TPC exhibited a nonlinear behavior with a distinct optimum within the tested range (Fig. 6). The highest TPC value (135.04 mg·100 g^−1^) was obtained at moderate ultrasound time (approximately 9 min) combined with short storage, attributable to controlled cell wall disruption and the release of phenolic constituents. The lowest TPC value (112.28 mg·100 g^−1^) was observed under short ultrasound time and long storage, indicating insufficient extraction and oxidative degradation of phenolics. The control samples exhibited a TPC of 83.8 mg·100 g^−1^ on day 16, corresponding to approximately 28.5 mg·100 g^−1^ lower phenolic content than the ultrasound-treated samples (∼34% relative increase). These results demonstrate that ultrasound time enhances phenolic release, whereas prolonged storage reduces it.

Increasing ultrasound time significantly enhanced TPC, with up to a 14.3% increase observed during extended storage (16 days) (Fig. 6). This effect was attributed to cavitation-induced increases in membrane permeability and the facilitated release of phenolic compounds, consistent with earlier studies [30], [44]. The molecular mechanism involves cavitation bubble formation and collapse, which induce partial matrix disintegration and release phenolics. In long-storage samples, a weakened matrix further strengthened the ultrasound effect. These findings agree with earlier studies [12], [38] and demonstrate that longer ultrasound exposure not only improves phenolic extraction but also partially offsets the degradative effects of extended storage.

The gradual decrease in TPC with increasing storage duration under both ultrasound conditions was attributed to oxidation, polymerization, and decarboxylation mechanisms, consistent with previous reports [34], [45]. The reduction was more pronounced under short-time ultrasound (13.8%), as incomplete phenolic release increased susceptibility to oxidative degradation [46], [47]. Longer ultrasound treatment promoted more complete extraction and greater phenolic stability, aligning with studies reporting enhanced extraction efficiency under optimal ultrasound conditions [48]. Recent studies further confirm that ultrasound-assisted extraction significantly enhances the recovery of total phenolic compounds in pomegranate and other plant matrices, while prolonged storage without treatment can reduce TPC; optimized sonication times and conditions (30–40 min, moderate power) maximize extraction and maintain phenolic content during storage [12], [49]. These results indicate that the interaction between ultrasound intensity and storage duration determines the extent of phenolic degradation; the initial extraction level and structural sensitivity of phenolics to oxidation explain the observed decline.

### 3.6 TAC

**Fig. 7:**
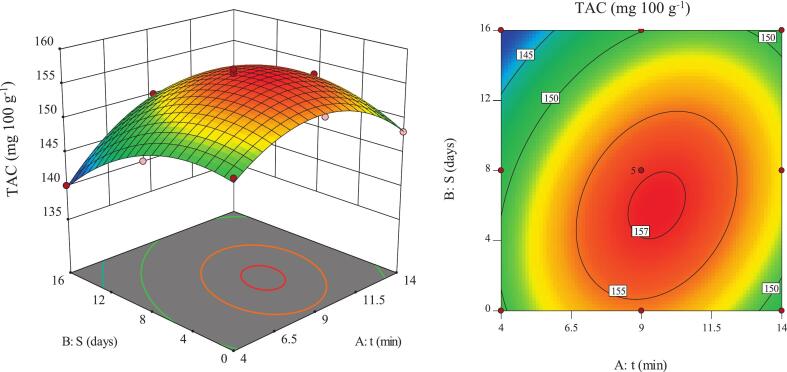
Two-dimensional and three-dimensional representation of the effects of ultrasound time and storage duration on the TAC of pomegranate arils.

TAC is a major indicator of flavonoid-based pigments with antioxidant properties in fruits and agricultural products and reflects the product’s capability to protect cells against oxidative damage while maintaining nutritional quality [21]. Data analysis showed that the interaction between ultrasound time and storage duration significantly affected the TAC of pomegranate arils (Fig. 7). The highest TAC value (157.15 mg·100 g^−1^) occurred at moderate ultrasound time combined with moderate storage duration, likely due to controlled cavitation and effective release of anthocyanins from the cellular matrix. In contrast, the lowest value (140.14 mg·100 g^−1^) was recorded under short ultrasound exposure and long storage, indicating progressive thermal and enzymatic degradation of anthocyanins. The control samples exhibited a TAC of 122.3 mg·100 g^−1^ on day 16, corresponding to approximately 17.8 mg·100 g^−1^ lower anthocyanin content than the ultrasound-treated samples (∼15% relative increase).

The effect of storage duration on TAC followed a biphasic trend (Fig. 7). During short storage durations, TAC increased, likely due to gradual release of remaining anthocyanins and postharvest enzymatic reactions [50]. With extended storage, TAC declined mainly due to chemical degradation and oxidation, especially under short ultrasound treatments and in the presence of oxygen or pH variations. Longer ultrasound exposure slowed this degradation by reducing the activity of anthocyanin-degrading enzymes [51], [52]. These outcomes are consistent with previous research on fruits and processed products, indicating that anthocyanin stability strongly depends on the initial release induced by physical treatments and storage conditions [12], [53].

Overall, the effect of ultrasound time on TAC also exhibited a biphasic pattern (Fig. 7). An initial increase in ultrasound time enhanced TAC through cavitation-induced release of anthocyanins [54]. However, exceeding the optimum duration resulted in TAC reduction due to partial degradation of anthocyanin structures and increased oxidation associated with free radical formation and localized heating [34]. During prolonged storage, the magnitude of TAC changes was lower, as long-term degradative reactions moderated the initial positive effect of ultrasound [38], [50]. The observed increase in TAC with moderate ultrasound time is in agreement with studies on other fruits, where short- to medium-duration ultrasound enhanced anthocyanin content and antioxidant capacity, while longer treatments or extended storage led to degradation [12], [28]. These results demonstrate that the early increase in TAC is governed by efficient cellular release, whereas the subsequent decline is driven by degradation and oxidation, emphasizing the importance of optimizing ultrasound time to preserve phenolic compounds during storage.

### 3.7 WL

**Fig. 8:**
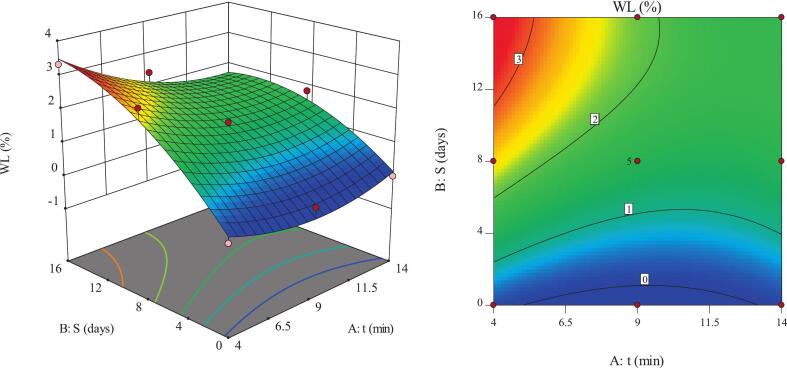
Effect of ultrasound time and storage duration on the WL of pomegranate arils.

The percentage of WL in fruits and horticultural products is regarded as a quantitative indicator for assessing the extent of water evaporation and tissue respiration, thereby reflecting changes in the physical and economic quality of the product during storage. This index is also used to compare the effects of postharvest treatments on freshness retention and to evaluate the role of packaging in minimizing damage [55]. The response surface plot indicated that the WL of pomegranate arils was significantly influenced by the cumulative effect of storage duration and, to a lesser extent, by ultrasound time (Fig. 8). The highest WL (3.32%) was observed under short ultrasound exposure combined with prolonged storage, suggesting the dominance of respiratory activity, evaporation, and moisture migration during extended storage durations. The lowest WL (0%) occurred at the onset of storage, when mass-transfer processes and cell-structure deterioration had not yet been activated. On day 16, the control samples exhibited a WL of 4.1%, representing approximately 0.78 percentage points higher than the ultrasound-treated samples (∼19% relative decrease), indicating that ultrasound effectively mitigated water loss during prolonged storage. This pattern confirms that storage duration is the primary driver of WL, while ultrasound serves as a moderating factor that can reduce the magnitude of this phenomenon only when it induces effective microstructural modifications.

An increase in ultrasound time resulted in a substantial reduction in WL (Fig. 8). This effect reflects the influence of ultrasound on cell membrane permeability and epidermal structure. Cavitation energy generated by ultrasound is likely to activate cellular defense mechanisms and stimulate biofilm synthesis, which enhances tissue resistance against evaporation and transpiration. The WL-reducing effect was more pronounced during extended storage (42.7% reduction), indicating the protective efficiency of ultrasound against chronic environmental stress and the reorganization of polymeric cell-wall networks [12], [13]. These outcomes align with previous findings on ultrasound applications in fruits and food matrices, which have shown that longer treatments enhance structural stability, reduce moisture loss, and preserve bioactive compounds, although the magnitude of effects depends on treatment parameters and the nature of the material [56], [57].

A direct increase in WL with longer storage duration was observed (Fig. 8). This trend results from continuous moisture evaporation and the consumption of carbon reserves through respiration, leading to greater cell-structural vulnerability over time [58], [59]. The upward trend was more pronounced in samples subjected to short ultrasound treatments, as moisture-loss pathways remained active and cellular matrices were not sufficiently reinforced. In contrast, prolonged ultrasound pretreatment exerted an opposite effect by stabilizing cell structure and reducing membrane permeability, thereby slowing the rate of WL. This finding highlights the capability of ultrasound to mitigate the adverse effects of long-term storage [13]. The observed reduction in WL with longer ultrasound treatment is consistent with other studies on fruits and food matrices, where ultrasound improved moisture retention, stabilized cell structures, and decreased weight loss during storage [13], [60]. These results are consistent with earlier studies on diverse fruits and food products, which reported that although storage extension invariably increases WL, ultrasound treatments effectively control this loss and maintain moisture stability [34], [61].

### 3.8 Optimization of parameters and practical validation of the model

**Fig. 9:**
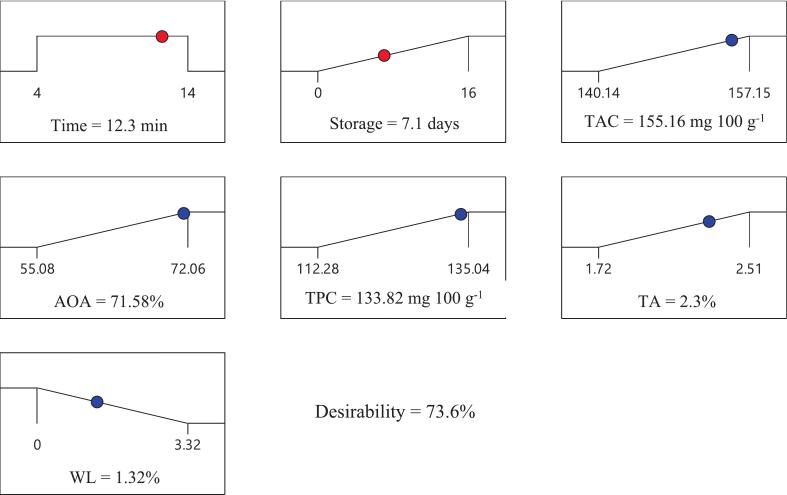
Effect of optimal ultrasound time and storage duration on biochemical and physiological properties of pomegranate arils.

In the multivariate optimization process using RSM and the desirability function, an ultrasound time of 12.3 min and a storage duration of 7.1 days were identified as the optimal conditions (Fig. 9). At this point, the model predicted maximum bioactive indices, including AOA (71.58%), TA (2.30%), TAC (155.16 mg·100 g^−1^), and TPC (133.82 mg·100 g^−1^), along with the minimum WL (1.32%). The overall desirability value of 73.6% indicated that the RSM model successfully balanced the maximization of bioactive compounds with the minimization of physical deterioration (Table 3).

**Table 3:** Comparison of predicted and actual data under optimal conditions.

Parameters	Selective criteria	Predicted results	Validation
t (min)	In range	12.3	12.3
S (days)	Maximum	7.1	7.1
AOA (%)	Maximum	71.58	71.06 ± 3.09
TA (%)	Maximum	2.3	2.37 ± 0.01
TAC (%)	Maximum	155.16	153.32 ± 7.97
TPC (mg 100 g^−1^)	Maximum	133.82	135.69 ± 6.26
WL (mg 100 g^−1^)	Minimum	1.32	1.31 ± 0.01
Desirability: 73.6%			

Experimental validation further confirmed the efficiency of the model; actual values included 71.06% for AOA, 153.32 mg·100 g^−1^ for TAC, 135.69 mg·100 g^−1^ for TPC, 2.37% for TA, and 1.31% for WL. Deviations of less than 3.2% between predicted and actual values demonstrated the high accuracy and reliability of the model. The close agreement between experimental and predicted data validated the precision and robustness of the RSM model in predicting variable behavior and in designing operational ultrasound conditions for improving the quality and shelf life of pomegranate arils.

This study demonstrated that the combined use of response surface methodology and the desirability function enables the determination of optimal ultrasound treatment conditions while preserving the maximal biochemical and physicochemical attributes of pomegranate arils. The findings are consistent with previous research on optimizing forage maize performance [15], carotenoid extraction from horticultural by-products [62], the effect of ultrasound on date fruit shelf life [17], and the optimization of coating treatments for mulberry quality [20]. This consistency strengthens the applicability and reliability of RSM in managing postharvest processes and enhancing the quality of horticultural crops.

## 4 Conclusion

The results of this study demonstrated that optimization of ultrasound treatment with appropriate time and storage duration is an effective strategy for preserving the biochemical and physicochemical properties of pomegranate arils during storage. Ultrasound application notably maintained bioactive compounds and limited weight loss. Compared to control samples, ultrasound-treated arils showed higher antioxidant activity, phenolic content, and reduced water loss, confirming the treatment’s superiority in preserving fruit quality. Multi-response modeling using response surface methodology (RSM) enabled identification of optimal process conditions and highlighted the critical role of storage duration on aril quality. Optimal conditions were 12.3 min ultrasound and 7.1 days storage with a desirability of 73.6%. The use of ultrasound can support practical postharvest management strategies and enhance product quality and shelf life. These findings emphasize the significance of mild non-thermal technologies in preserving the quality of sensitive food products, while future research may explore packaging effects and extended storage conditions.

## CRediT authorship contribution statement

**Isa Hazbawi:** Writing – review & editing, Writing – original draft, Visualization, Validation, Supervision, Software, Resources, Project administration, Methodology, Investigation, Formal analysis, Data curation. **Hamed Etezadi:** Writing – review & editing, Writing – original draft, Visualization, Software, Resources, Methodology, Formal analysis.

## Declaration of competing interest

The authors declare that they have no known competing financial interests or personal relationships that could have appeared to influence the work reported in this paper.
